# Chloride/Multiple Anion Exchanger SLC26A Family: Systemic Roles of SLC26A4 in Various Organs

**DOI:** 10.3390/ijms25084190

**Published:** 2024-04-10

**Authors:** Dongun Lee, Jeong Hee Hong

**Affiliations:** Department of Health Sciences and Technology, GAIHST (Gachon Advanced Institute for Health Sciences and Technology), Lee Gil Ya Cancer and Diabetes Institute, Gachon University, 155 Getbeolro, Yeonsu-gu, Incheon 21999, Republic of Korea; sppotato1@gmail.com

**Keywords:** SLC26A transporters, SLC26A4, anion exchanger, bicarbonate transporters

## Abstract

Solute carrier family 26 member 4 (SLC26A4) is a member of the SLC26A transporter family and is expressed in various tissues, including the airway epithelium, kidney, thyroid, and tumors. It transports various ions, including bicarbonate, chloride, iodine, and oxalate. As a multiple-ion transporter, SLC26A4 is involved in the maintenance of hearing function, renal function, blood pressure, and hormone and pH regulation. In this review, we have summarized the various functions of SLC26A4 in multiple tissues and organs. Moreover, the relationships between SLC26A4 and other channels, such as cystic fibrosis transmembrane conductance regulator, epithelial sodium channel, and sodium chloride cotransporter, are highlighted. Although the modulation of SLC26A4 is critical for recovery from malfunctions of various organs, development of specific inducers or agonists of SLC26A4 remains challenging. This review contributes to providing a better understanding of the role of SLC26A4 and development of therapeutic approaches for the SLC26A4-associated hearing loss and SLC26A4-related dysfunction of various organs.

## 1. Introduction

Transporters encoded by the *SLC26* gene are anionic transporters [1]. SLC26 transporters contain various subfamilies, including SLC26A1 and SLC26A11 [1]. SLC26 transporters contain 12 transmembrane domains and a sulfate transporter and an anti-sigma factor antagonist (STAS) domain in the C-terminal cytoplasmic region [1]. Each transporter has a predominant substrate [1]. SLC26A1, also known as Sat-1, transports SO_4_^2−^, oxalate, and glyoxylate and is mainly expressed in hepatocytes, renal proximal tubules, and intestines [2,3]. SLC26A2, also known as diastrophic dysplasia sulfate transporter (DTDST), transports SO_4_^2−^, oxalate, and Cl^−^ and is mainly expressed in chondrocytes, renal proximal tubules, intestines, and pancreatic ducts [4]. SLC26A3 is downregulated in adenoma (DRA) and chloride-losing diarrhea (CLD), transports Cl^−^, HCO_3_^−^, and oxalate, and is mainly expressed in enterocytes, sperm, and epididymis [5,6]. SLC26A4 (pendrin) transports I^−^, Cl^−^, HCO_3_^−^, and SCN^−^ and is broadly expressed in cochlear cells, vestibular epithelial cells, thyrocytes, type B intercalated cells, and airway epithelial cells [7,8]. SLC26A5, also known as prestin, transports Cl^−^, formate, oxalate, and SO_4_^2−^ and is mainly expressed in cochlear hair cells [9,10]. SLC26A6, also known as Pat-1 and Cl^−^/formate exchanger (CFEX), transports Cl^−^, HCO_3_^−^, oxalate, OH^−^, and formate and is broadly expressed in enterocytes, pancreatic ducts, renal proximal tubules, cardiac myocytes, and sperm [3,11,12]. SLC26A7, also known as SUT2, transports Cl^−^, HCO_3_^−^, and OH^−^ and is broadly expressed in gastric parietal, type A intercalated, and endothelial cells [13,14]. SLC26A8, also known as TAT1, transports Cl^−^ and SO_4_^2−^ and is mainly expressed in male germ cells and sperm [15,16]. SLC26A9 transports Cl^−^ and HCO_3_^−^ and is mainly expressed in airway epithelial cells and gastric parietal cells [17]. SLC26A10 is a pseudogene that is not included in the human open reading frame [1]. SLC26A11, also known as SUT1 and KBAT, transports Cl^−^, HCO_3_^−^, SO_4_^2−^, and oxalate and is broadly expressed in renal intercalated cells, pancreatic ducts, endothelial cells, and the brain [18,19]. The representative characteristics of SLC26A transporters are summarized in Table 1.

SLC26A4 transports different anions, including I^−^, Cl^−^, HCO_3_^−^, and SCN^−^, depending on the organ type [20]. SLC26A4 exchanges Cl^−^ and HCO_3_^−^ in endolymphatic sac epithelial cells, such as the inner ear and type B intercalated cells, and transports I^−^ in follicular cells, such as thyroid cells [20]. Furthermore, cryo-EM shows the symmetric homodimer of SLC26A4 in the presence of Cl^−^ in immortalized human embryonic kidney (HEK293E) cells [20]. SLC26A4-mediated ion transportation is modulated by the STAS domain, which forms a long loop region [20]. STAS promoters induce SLC26A4 dimerization [21]. Additionally, the misfolded SLC26A4 through mutation is recovered by the STAS domain [22]. SLC26A4 is a well-known cause of inherited diseases, including autosomal recessive non-syndromic deafness, DFNB4, and Pendred syndrome [23]. Thus, the symptoms, especially hearing loss, are presented at a young age. In this respect, treatment of SLC26A4 has been attempted for patients during childhood with cochlear implantation [24,25]. Mutations in *SLC26A4* have been identified in patients with autosomal recessive non-syndromic deafness (DFNB)4 and Pendred syndrome with hearing loss [26]. The mutation of SLC26A4 induces loss of ion transportation such as Cl^−^, HCO_3_^−^, and I^−^ in SLC26A4-mutated organs, including the cochlea and inner ear [27]. Most studies on SLC26A4 have focused on the relationship between hearing loss and *SLC26A4* mutations and have been extensively reviewed elsewhere. Additionally, most studies on SLC26A4 in ear tissues have focused on gene mutations. Therefore, to understand the pathophysiological roles of SLC26A4, we discussed the overall roles of the SLC26A family. In this review, we demonstrated the physiological and pathological mechanisms of SLC26A4, including other SLC26A family members, with a view toward immune and systemic regulation in various organs other than the ear. In Section 5, although we summarized the physiological role of other organs than the ear, we suggest therapeutic approaches to recover expression of SLC26A4, which is mutated in SLC26A4-related hearing loss. This is because recent studies on SLC26A4-related diseases have mostly focused on hearing loss. The understanding presented by these recent studies regarding hearing loss suggests the potential to treat physiological SLC26A4-mediated dysfunction in other organs.

## 2. Multiple Physiological Functions of SLC26A4

### 2.1. Protection of SLC26A4 in Airway Epithelium

**Figure 1:**
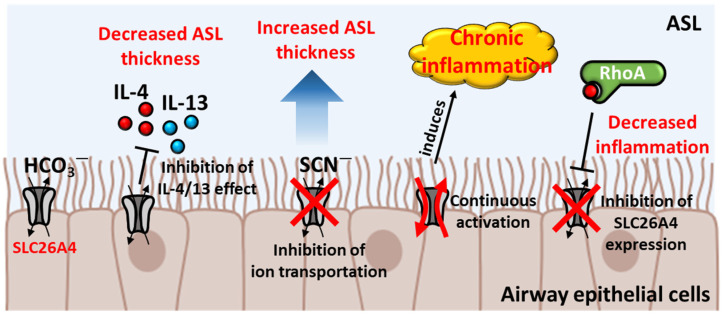
Role of SLC26A4 in airway epithelial cells. Schematic illustration of the physiological roles of SLC26A4 in airway epithelial cells. The transportation of HCO_3_^−^ through SLC26A4 maintains ASL thickness, and abnormal activation of SLC26A4 induces chronic inflammation, which is inhibited by RhoA.

The airway epithelium encounters antigens that enter the airway through respiration [28]. The airway epithelium removes and neutralizes harmful external substances [28]. Thus, the functional maintenance of airway epithelial cells is critical for protecting the body. In this section, we summarize the relationship between SLC26A4 and the airway epithelium, which contributes to respiratory inflammation (Figure 1).

The airway epithelium contributes to immune response by blocking external components such as particles and inactivating infectious materials through airway surface liquid (ASL) [29,30,31]. ASL is a thin layer or fluid with an acidic pH, and a defect in the pH modulation of ASL causes respiratory diseases [32]. The regulation of pH is a key factor in protecting the airway epithelium. In this respect, HCO_3_^−^ transportation through SLC26A4 plays a critical role in protecting the airway epithelium and its immune response. ASL is thickened by allergic cytokine interleukin-4/13 (IL-4/13), and the efflux of HCO_3_^−^ from epithelial cells through SLC26A4 decreases the ASL thickness [33,34]. The secreted HCO_3_^−^ is transformed into H_2_CO_3_ and then converted to H_2_O and CO_2_ by the regulation of carbonic anhydrase in ASL [34]. Increased H_2_O decreases osmotic pressure and then lowers ASL thickness [34]. The nasal epithelium of patients with non-syndromic hearing loss (DFNB4) is thicker than that of healthy individuals [35]. IL-13 treatment induces additional ASL thickness in the DFNB4 nasal epithelium compared with that in the normal nasal epithelium [35]. The IL-13-induced HCO_3_^−^ transportation by SLC26A4 in DFNB4 nasal epithelial cells is lower than that in the normal nasal epithelium [35]. In addition, inhibition of SCN^−^ transportation induces ASL thickness in primary human bronchial epithelial cells [36]. Treatment of PDSinh-A01, an SLC26A4 inhibitor, enhances IL-13-induced thickness of ASL in primary cultured human bronchial epithelial cells [36]. These results suggest that SLC26A4 activity is involved in the modulation of ASL thickness. To protect the airway by inducing an immune response, the expression of SLC26A4 is increased and SLC26A4 is localized to the plasma membrane through IL-4 and IL-13 stimulation in human bronchial epithelial cells [37,38]. Continuous activation of SLC26A4 induces chronic respiratory inflammation, such as asthma, by producing inflammatory factors including NF-kB, IL-33, and thymic stromal lymphopoietin [39,40]. To modulate this exaggerated inflammatory response, airway epithelial cells induce the Ras homolog family member A (RhoA) inhibitory pathway [40]. The activation of RhoA inhibits Slc26a4 expression and induces TGF-β1 expression, which inhibits Slc26a4-induced inflammation in mouse type 2 alveolar epithelial cells [40]. Deletion of *RhoA* increases inflammatory cytokine levels in asthma mouse models [40]. In addition, SLC26A4 is associated with lipopolysaccharide (LPS)-induced lung injury [41,42]. LPS injection increases the expression of inflammatory cytokines and Slc26a4 in C57BL/6 mice [41]. Deletion of *Slc26a4* attenuates LPS-induced NF-kB activation and lung injury in mice [42].

### 2.2. Regulation of Blood Pressure

**Figure 2:**
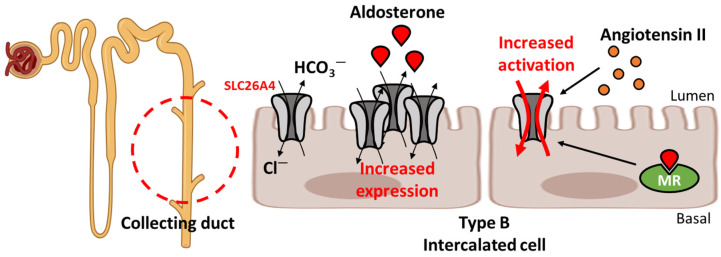
Relationship between hormonal reactions and SLC26A4 expression in the kidney. SLC26A4 is activated by aldosterone and angiotensin II in type B intercalated cells to transport Cl^−^ and HCO_3_^−^. Increased activation of SLC26A4 induces high blood pressure. MR: mineralocorticoid receptor.

The kidneys are key organs that regulate fluid volume and blood pressure [43]. The kidneys transport ions, including sodium, potassium, and chloride, with aldosterone-induced hormonal reactions initiated by renin and angiotensin II [44,45]. Among the various ion channels and transporters in the kidney, SLC26A4 plays a prominent role [45,46,47] (Figure 2). SLC26A4 is expressed in aldosterone-sensitive regions, including the distal convoluted tubule, connecting tubule, and cortical collecting duct [48,49,50]. For instance, in the collecting duct, SLC26A4 is involved in Cl^−^/HCO_3_^−^ exchange in the apical membrane of intercalated cells [48,49,50]. Slc26a4 is upregulated by aldosterone stimulation in mouse type B intercalated cells and increases blood pressure [51,52]. Aldosterone increases the apical expression of Slc26s4 in the cortical collecting duct, whereas the deletion of *Slc26a4* attenuates aldosterone-induced Cl^−^/HCO_3_^−^ exchange activity in the lumen of type B intercalated cells [51]. Additionally, aldosterone induces Cl^−^ absorption, whereas deletion of *Slc26a4* inhibits Cl^−^ absorption and HCO_3_^−^ secretion in the cortical collecting ducts of mice with lower blood pressure [52]. In a clinical report, the blood pressure of patients with *SLC26A4* mutations was lower than that of the normal group [53]. The patients with *SLC26A4* mutations showed increased excretion of urinary Na^+^ and Cl^−^, and the levels of serum renin and angiotensin II in these patients were higher than those in the normal group [53]. In addition, lower blood pressure was observed with the deletion of the *Slc26a4* gene in a mouse model than in wild-type mice [54]. The deletion of *Slc26a4* increases concentration of HCO_3_^−^, whereas it decreases the concentrations of Na^+^ and Cl^−^ in mouse blood, suggesting that slc26a4 modulates blood pressure-associated electrolyte levels [54]. In addition to aldosterone, angiotensin II increases SLC26A4 activity through aldosterone coactivation in the kidneys [55]. Slc26a4 is stimulated by the activation of mineralocorticoid receptor (MR), which binds aldosterone and is activated by angiotensin II treatment in mouse intercalated cells [56,57]. Aldosterone and angiotensin II induce Slc26a4 expression, whereas MR deletion decreases Slc26a4 expression in the apical membrane of type B intercalated cells [56,57]. Co-administration of aldosterone and angiotensin II increases Slc26a4 expression and Na^+^/Cl^−^ reabsorption in adrenalectomized mouse kidneys, which are adrenal glands removed from a mouse model [58]. Angiotensin II increases Cl^−^ reabsorption through Slc26a4 and induces sodium chloride cotransporter (NCC) activation to increase Na^+^ reabsorption in adrenalectomized mouse kidneys [58]. In addition, the E3 ubiquitin ligase, Nedd4-2, which is inhibited by the aldosterone-induced MR pathway, downregulates epithelial sodium channel (ENaC) and pendrin activity [59]. Deletion of *Nedd4-2* increases the Cl^−^/HCO_3_^−^ exchange activity and pendrin expression in the apical membrane of mouse type B intercalated cells [59]. SLC26A4 expression is associated with the movement of ions, such as Na^+^ and Cl^−^ reabsorption. Thus, SLC26A4 is a critical component of blood pressure regulation in the kidneys.

### 2.3. Involvement in Hormone Regulation

The thyroid gland regulates various metabolic processes in the body, including bone formation, mitochondrial biogenesis, and nutrient (protein, carbohydrate, and lipid) metabolism through hormones [60,61]. Thyroid hormones stimulate various target tissues, including the heart, brain, bones, and muscles [62]. I^−^ is considered as a major component of thyroid hormones, and I^−^ transportation through SLC26A4 is essential for the thyroid gland [63]. SLC26A4-mediated ion transportation is focused on I^−^/HCO_3_^−^ transportation in the thyroid [64,65,66]. The thyroid-stimulating hormone (TSH) stimulates I^−^ efflux via Slc26a4 in rat thyroid PCCL-3 cells [67]. In addition, TSH stimulates translocation of Slc26a4 to the plasma membrane [67]. Malfunction of the thyroid gland induces overproduction of thyroid hormones, known as hyperthyroidism, or lower production of thyroid hormones, known as hypothyroidism. A recent study showed that dual oxidases are the major components of hydrogen peroxide generation, which induces hormone synthesis in the thyroid gland [68]. Dual oxidase expression is stimulated by IL-4 and the Janus kinase1/signal transducer and activator of transcription 6 pathway [68]. Overexpression of IL-4 induces hyperthyroidism and increases SLC26A4 expression [69,70]. In overexpressed-Il-4 transgenic mice, mRNA expression of Duox1, which is a marker of thyroid hormonal function, and protein expression of Slc26a4 are increased [69]. The serum concentration of TSH is increased in Il-4 transgenic mice, and goiter development is enhanced in low iodine-fed mice [70]. Deletion of Slc26a4 enhanced the increase in TSH and goiter development in mice [70]. Additionally, patients with a goiter with accompanying hypothyroidism have a swollen thyroid and *SLC26A4* gene mutations [71]. Mutation-induced deficiency of *SLC26A4* induces hypothyroidism [72]. Excessive iodine intake induces overactivation of the thyroid gland and causes thyroid diseases, including hyperthyroidism [73]. Overconsumption of I^−^ in mice triggers a negative feedback-like signal that inhibits the activity of Slc26a4 to regulate the hyperactivation of the thyroid gland [74].

### 2.4. Other Tissues and Potential Negative Regulators of Tumors

In addition, as in the ear, as referred to in Section 1, SLC26A4 is associated with the nasal system [75]. SLC26A4 is mainly expressed in the epithelial membrane of turbinate mucosa and nasal polyps [75]. In clinical studies, patients with nasal polyps show increased SLC26A4 expression in eosinophilic chronic rhinosinusitis [76,77]. SLC26A4 plays a role not only in non-tumor cells but also in tumor cells. In MCF-7 breast cancer cells, SLC26A4 is expressed and transports I^−^ [78]. Additionally, treatment of carcinogen, N-methyl-N-nitrosourea, with I_2_ increases Slc26a4 expression in rat mammary glands [79]. These results suggest that the tumorigenic circumstance of breast cancer increases Slc26a4 expression. However, the mRNA and protein expression of SLC26A4 were lower in tumoral regions than in peri-tumoral regions in patients with breast cancer [80]. In addition, analysis of gene expression patterns showed that *SLC26A4* expression was downregulated in patients with prostate and thyroid cancers [81,82]. Thus, the expression patterns and location of SLC26A4 along with cancer types show potential for diagnosis of cancers. Moreover, it was shown that cell-free DNA, which has been suggested for cancer diagnosis and progression, of SLC26A4 is hypermethylated in blood of thyroid cancer patients [83]. It is well-known that DNA hypermethylation inhibits methylated gene expression [84,85]. Interaction of SLC26A4 and methylation should be determined in future study. Although decreased SLC26A4 expression is a common pattern in prostate, thyroid, and breast cancers, the relationship between SLC26A4 and tumors has not been fully demonstrated.

## 3. Relationship between SLC26A4 and Other Ion Transporters

In particular, the ion transport of SLC26A4 is different from that of cystic fibrosis (CF) transmembrane conductance regulator (CFTR) in various organs and tissues, including the lung, kidney, thyroid, inner ear, parotid duct, and liver [86]. SLC26A4 reabsorbs Cl^−^ and secretes HCO_3_^−^, whereas CFTR transports both Cl^−^ and HCO_3_^−^ outside the plasma membrane [86]. A correlation between SLC26A4 and CFTR has been demonstrated in several experimental systems. Thus, we described the relationship between SLC26A4 and CFTR. In addition to CFTR, relationships between SLC26A4 and other ion channels/transporters are discussed in this section.

IL-4 and IL-13 activate SLC26A4 and CFTR in CF airway epithelial cells [87]. In addition, IL-4/IL-13-induced CFTR activation is attenuated by treatment with SLC26A4 inhibitor niflumic acid [87]. CFTR mutations have no effect on pH-related proteins such as H^+^/K^+^ ATPase (ATP12A) and sodium bicarbonate cotransporter 1 (SLC4A4); however, changes in pH through *SLC26A4* modulation are inhibited by CFTR mutations in human bronchial epithelial cells [88]. SLC26A4 is activated by treatment with CFTR inducer forskolin [89]. These two proteins are closely related to CF, a hereditary disorder of human airway epithelial cells [90,91]. In patients with CF, the 723rd histidine of SLC26A4 is converted to arginine and the 508th phenylalanine of CFTR is deleted [90,91]. Thus, to rescue these misfolded proteins, endoplasmic reticulum (ER) stress-mediated secretion and the ubiquitin–proteasome system are considered useful strategies for deleting mutated SLC26A4 and CFTR [91,92,93,94]. These therapeutic mechanisms are described in Section 5.

CFTR deficiency induces an acid–base imbalance caused by the deactivation of SLC26A4 [86]. CFTR is localized to the Slc26a4-positive membrane of the mouse cortical collecting duct [95]. Deletion of *Cftr* attenuates HCO_3_^−^ excretion from the mouse kidneys [96]. The knockout of *Cftr* decreases urine pH levels while increasing serum pH levels in mice [97]. Expression of *Slc26a4* mRNA and Slc26a4 protein is decreased by Cftr knockout in mouse kidneys [97]. Na^+^ is a major ion that regulates blood pressure in the kidney through Na^+^ channels such as ENaC [98,99]. The relationship between SLC26A4 and ENaC has been studied extensively. SLC26A4-induced increases in HCO_3_^−^ and pH levels stimulate ENaC activity [47,100,101]. Activation of Slc26a4 increases Enac expression, whereas knockout of *Slc26a4* reduces Enac-mediated Na^+^ absorption [101]. Another Na^+^ transporter, NCC, interacts with SLC26A4 [102]. The expression of Slc26a4 is compensatorily increased by NCC knockout in mice [102].

In addition to Na^+^, K^+^ is a key ion that regulates blood pressure [103,104]. A K^+^-free diet decreases Slc26a4 expression [105], whereas extreme restriction of K^+^ in the diet increases SLC26A4 expression in mouse kidneys [106]. The activation of Slc26a4 through aldosterone stimulation induces hypokalemia in the plasma, whereas addition of K^+^ recovers the concentration of plasma K^+^ [105].

## 4. Role of Other SLC26A Transporters with SLC26A4

**Figure 3:**
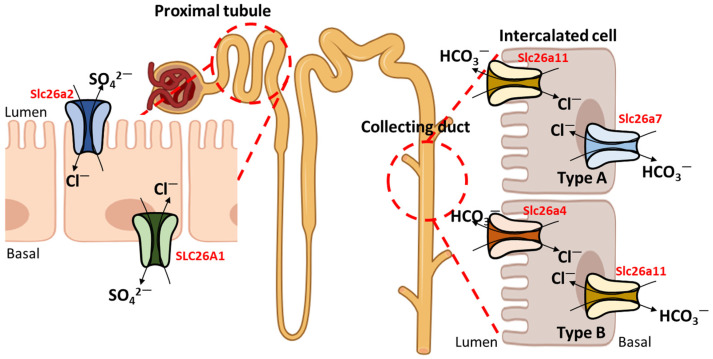
Distribution of SLC26A transporters. SLC26A transporters are distributed in the kidney cells. SLC26A1 (basal membrane) and Slc26a2 (luminal membrane) are expressed in proximal tubular cells. Slc26a7 (basal membrane) and Slc26a11 (luminal membrane) are expressed in type A intercalated cells. Slc26a11 (basal membrane) and Slc26a4 (luminal membrane) are expressed in type B intercalated cells.

As members of the SLC26A family contain common structures, including the cytoplasmic N-terminal domain followed by 12 transmembrane domains, and transport common ions, including HCO_3_^−^, Cl^−^, and I^−^, SLC26A transporters show potential for crosstalk with each other [1]. For example, SLC26A1 transports Cl^−^ and SO_4_^2−^ in the basolateral membrane of the proximal tubular kidney cell line (LLC-PK1), whereas Slc26a2 transports Cl^−^ and SO_4_^2−^ in the apical membrane of rat proximal tubules [107,108]. Similarly, Slc26a7 exchanges HCO_3_^−^ and Cl^−^ in the basolateral membrane of rat type A intercalated cells, whereas Slc26a11 transports HCO_3_^−^ and Cl^−^ in the apical membrane of mouse type A intercalated cells [13,109]. Slc26a4 transports HCO_3_^−^ and Cl^−^ in the apical membrane of mouse type B intercalated ducts, whereas Slc26a11 transports HCO_3_^−^ and Cl^−^ in the basolateral membrane of mouse type B intercalated ducts [49,109]. The schematic relationships between these transporters are shown in Figure 3. Although they possess various common characteristics, their relationships have not been fully studied. In this section, we highlight the role of other SLC26A transporters in various tissues (Table 2).

In the SLC26A family, SLC26A9 is the most studied transporter in the airways. In addition to SLC26A4, SLC26A9 is expressed in the apical membrane of airway epithelial cells in humans, mice, and piglets [117,119]. Transportation of HCO_3_^−^ through Slc26a9 induces acidification of ASL in mice [117]. Additionally, in the human lung bronchiolar and alveolar epithelium, SLC26A9 transports Cl^−^, suggesting that it is a Cl^−^ channel [122,123]. SLC26A9 performs CFTR-like functions in the airways and interacts with CFTR [124]. Inhibition of CTFR through CFTR inhibitor GlyH-101 decreases the SLC26A9 current in CFTR or SLC26A9-overexpressed HEK293 cells and in human bronchial epithelial (HBE) cells [122,125]. Co-overexpression of SLC26A9 and CFTR in HEK293 cells reduces forskolin (cAMP activator)-induced CFTR currents compared with CFTR-only transfected HEK293 cells [123]. Overexpression of SLC26A9 increases the current in a *CFTR*-mutated (ΔF508, deletion of phenylalanine) HBE cell line (CFBE41o) [118]. Because ΔF508 CFTR represents CF, SLC26A9 has been suggested as a therapeutic target for CF [126,127]. Additionally, SLC26A9 is associated with asthma and lung inflammation. In the asthmatic airways of humans, SLC26A9 is overexpressed and genetic variants of *SLC26A9* increase risk of asthma [119,120]. Although other SLC26A transporter genes, including *SLC26A3*, *SLC26A6*, and *SLC26A9*, are expressed in HBE cells, and SLC26A3 transports Cl^−^ and HCO_3_^−^ in tracheal epithelial cells [128,129], the detailed regulatory mechanisms and physiological roles of other SLC26A family members have not been fully studied.

Several SLC26A transporters contribute to the regulation of blood pressure. Regulatory hormone vasopressin increases Slc26a7 expression in the renal outer medulla of rats for water reabsorption and subsequent increase in blood pressure [113]. Additionally, Slc26a7 is upregulated by K^+^ depletion in the renal outer medulla of rats and mice [112,113]. Although direct evidence of the regulation of blood pressure through SLC26A7 has not yet been demonstrated, SLC26A7 expression is increased by K^+^ depletion-induced high blood pressure. Depletion of K^+^ increases blood pressure in serial steps in the basolateral membrane of the distal convoluted tubule [130]. A low concentration of potassium increases K^+^ transportation through the inwardly rectifying potassium channel (Kir) 4.1/5.1 on the kidney basolateral membrane, and K^+^ transportation subsequently induces membrane hyperpolarization [130]. The membrane hyperpolarization induces Cl^−^ transportation to decrease the cytosolic Cl^−^ concentration, and NCC is activated to increase Na^+^ reabsorption and subsequently enhance blood pressure [130]. SLC26A9 affects the regulation of blood pressure. Deletion of *Slc26a9* reduces Cl^−^ transportation in the mouse kidney medullary collecting duct and increases arterial and blood pressure in mice [121].

In the inner ear, Slc26a4 regulates oxalate transportation, and mutations in *Slc26a4* generate calcium oxalate stones in the inner ear [131]. In the kidney, other SLC26A transporters also regulate oxalate concentration. Among the SLC26A family members, SLC26A6 is a representative transporter of oxalate. Deletion of *Slc26a6* attenuates Cl^−^/oxalate exchange in mouse proximal tubules and increases the concentration of mouse urine oxalate [110]. Additionally, knockout of *Slc26a6* induces calcium oxalate stones in the mouse bladder [110]. Kidney stones are associated with both SLC26A6 and estrogen levels. Estrogen inhibits the generation of kidney stones, and malfunction of SLC26A6 generates kidney stones through systemic reviews and meta-analyses in female patients [132]. Estrogen activates SLC26A6 in the kidneys, and estrogen-deficient females show lower SLC26A6 activation, with an increase in kidney stones [132]. SLC26A6 regulates not only oxalate transportation but also the maintenance of renal pH. Deletion of *Slc26a6* decreases pH, with a decrease in sodium hydrogen exchanger 3 (Nhe3) in mouse proximal tubule cells [111]. Similarly, deletion of *Slc26a7* induces distal renal tubular acidosis in mice [114]. Overexpression of Slc26a7 increases pH in Madin–Darby canine kidney (MDCK) cells, and acidification of the culture media decreases Slc26a7 expression in MDCK cells [133].

Patients with goitrous hypothyroidism harbor *SLC26A4* and *SLC26A7* mutations [134]. Knockout of *Slc26a7* decreases the concentration of thyroid hormones and abnormally increases the size of the mouse thyroid gland [115]. Additionally, TSH induces the translocation of *Slc26a7* from the cytosol to the plasma membrane in rat thyroid follicular FRTL-5 cells [116]. The thyroid hormone levels in *Slc26a7*-deleted mice are lower than those in *Slc26a4*-deleted mice [135]. Although SLC26A7 is associated with thyroid hormone regulation, the relationship between SLC26A4 and SLC26A7 in the thyroid gland has not yet been demonstrated. To fully understand the regulation of I^−^ transport, the physiological role of SLC26A7 and its interaction with SLC26A4 should be verified.

## 5. Therapeutic Approaches

The diseases that are related to SLC26A4 are generally induced by the dysfunction of *SLC26A4*. However, the development of specific inducers or agonists of SLC26A4 remains a challenging issue. As referred to in Section 2, aldosterone and angiotensin II increase SLC26A4 activity, whereas treatments with aldosterone and angiotensin II are restricted to inducer use for SLC26A4 because aldosterone and angiotensin II increase blood pressure. Although development of drugs targeting ion channels/transporters has been studied, it was limited to machine learning techniques [136]. Additionally, although a recent study presents a clinical trial to treat Pendred syndrome with sirolimus, which is an mTOR inhibitor, this study is still emerging [137]. A clinical trial of SLC26A4 mutation-induced hearing loss was suggested with a cochlear implant [138], whereas the studies of SLC26A4-related disease treatment are restricted to gene delivering therapy. In a recent study, hearing loss in *Slc26a4*-deficient mice was reversed by gene therapy, by inserting *SLC26A4* cDNA into *Slc26a4*-deficient embryonic mice [139]. Although viral transfection of *Slc26a4* induced insufficient restoration of vestibular function, hearing loss was recovered, cochlear enlargement was inhibited, and outer hair cells were rescued [139]. The splice site mutation in *SLC26A4*, which commonly occurs in Asian populations, induces Pendred syndrome with the deletion of exon 8 [140]. To rescue skipping exon 8, antisense oligonucleotides were used to promote exon inclusion [140]. Antisense oligonucleotides of *SLC26A4* recovered the length of *SLC26A4* in patients with *SLC26A4* mutations regarding the *Slc26a4* site-mutated mouse model [140]. Attempts to modulate *SLC26A4* have occurred; however, an experimental approach has been proposed for hearing loss [141]. The mutation of *SLC26A4*, p.H723R (His723Arg), induces misfolding of SLC26A4 and inhibits surface expression of SLC26A4 [142]. H723R-transfected HEK293 cells did not show Cl^−^/HCO_3_^−^ exchange activity compared with SLC26A4 wild-type transfected HEK293 cells [94]. The induction of unconventional protein secretion has been suggested as a treatment approach. Unconventional protein secretion is induced by the inhibition of ER-to-Golgi transport to rescue the trafficking of mutated ion transporters, including CFTR [93]. The inhibition of ER-to-Golgi transport by the dominant-negative form of ADP-ribosylation factor 1 recovered SLC26A4 activity with heat shock cognate protein 70 in H723R-transfected HEK293 cells [94]. Regulation of ion channels and transporters has been suggested for the treatment of various ion channel diseases as drug targets [143,144]. Numerous ion channels/transporters, including potassium, sodium, calcium, and chloride channels, are associated with diseases in various organs, including the brain, heart, pancreas, kidneys, and skeletal muscles [143]. For example, neurological and cardiac channelopathies occur due to malfunctions of various ion channels, such as voltage-gated sodium channels, voltage-gated calcium channels, GABA-gated chloride channels, glutamate-gated cationic channels, and acetylcholine-gated cationic channels [144]. However, therapeutic approaches for the treatment of SLC26A4-related gene mutations or defects have to be studied further with clinical trials.

## 6. Conclusions and Perspectives

In this review, we summarized the relationship between SLC26A4 and its pathophysiological functions in various organs. SLC26A4 is involved in the maintenance of airway pH, which induces ASL, lung inflammation, injury, and increased blood pressure. SLC26A4 deficiency is observed in hypothyroidism patients, whereas increased expression of SLC26A4 is observed in hyperthyroidism. However, despite their diverse regulatory mechanisms, the detailed mechanisms of SLC26A4 and the relationship between SLC26A4 and other SLC26A transporters need to be identified as potential challenging issues. In recent research, Cl^−^/HCO_3_^−^ exchangers including SLC26A6 and SLC4A2 (although not SLC26A transporter) were suggested as complementary to ion transportation for HCO_3_^−^ secretion in esophageal submucosal glands [145]. SLC26A6 secretes HCO_3_^−^ on the luminal membrane of mouse esophageal submucosal glands and SLC4A2 induces influx of HCO_3_^−^ from the basal membrane of mouse esophageal submucosal glands [145]. With regard to this regulation, SLC26A transporters in the basal membrane should be considered with mutual regulation of SLC26A4. In addition, therapeutic approaches for conjugated function could be considered a new challenge for future researchers. Recent research has demonstrated modulation of the SLC26A4 gene by CRISPR/Cas to cause congenital hearing loss [146]. Application of CRISPR/Cas techniques against mutations of SLC26A4 demonstrates the potential to recover congenital hearing loss. The development of modulatory drugs or genetic applications, such as CRISPR/Cas technology, and the induction of unconventional secretion of SLC26A4 are welcomed in SLC26A4-related diseases.

## Figures and Tables

**Table 1:** List of SLC26A transporter family members.

Gene Name	Protein Name	Transporting Ions	Expression	Refs.
*SLC26A1*	Sat-1	SO_4_^2−^, oxalate, glyoxylate	Hepatocyte, renal proximal tubule, intestine	[2,3]
*SLC26A2*	DTDST	SO_4_^2−^, oxalate, Cl^−^	Chondrocyte, renal proximal tubule, intestine, pancreatic duct	[4]
*SLC26A3*	DRA, CLD	Cl^−^, HCO_3_^−^, oxalate	Enterocyte, sperm, epididymis	[5,6]
*SLC26A4*	Pendrin	I^−^, Cl^−^, HCO_3_^−^, SCN^−^	Cochlear, vestibular epithelial cell, thyrocyte, type B intercalated cell, airway epithelial cell	[7,8]
*SLC26A5*	Prestin	Cl^−^, formate, oxalate, SO_4_^2−^	Cochlear hair cell	[9,10]
*SLC26A6*	Pat-1, CFEX	Cl^−^, HCO_3_^−^, oxalate, OH^−^, formate	Enterocyte, pancreatic duct, renal proximal tubule, cardiac myocyte, sperm	[3,11,12]
*SLC26A7*	SUT2	Cl^−^, HCO_3_^−^, OH^−^	Gastric parietal cell, type A intercalated cell, endothelial cell	[13,14]
*SLC26A8*	TAT1	Cl^−^, SO_4_^2−^	Male germ cell, sperm	[15,16]
*SLC26A9*	-	Cl^−^, HCO_3_^−^	Airway epithelial cell, gastric parietal cell	[17]
*SLC26A11*	SUT1, KBAT	Cl^−^, HCO_3_^−^, SO_4_^2−^, oxalate	Renal intercalated cell, pancreatic duct, endothelial cell, brain	[18,19]

Abbreviations: DTDST, diastrophic dysplasia sulfate transporter; DRA, downregulated in adenoma; CLD, Cl^−^-losing diarrhea; CFEX, Cl^−^/formate exchanger.

**Table 2:** The other SLC26A functions in various tissues and cells.

Transporters	Expression	Functions	Refs.
Slc26a6	Mouse bladder	Induction of calcium oxalate stones	[110]
	Mouse proximal tubule	Decrease in Nhe3 expression	[111]
SLC26A7, Slc26a7	Mouse renal outer medulla	Increased by high blood pressure	[112,113]
	Mouse distal renal tubule	Induction of acidosis	[114]
	Mouse thyroid	Decrease in thyroid hormone	[115]
	FRTL-5	Translocated by thyroid stimulating hormone	[116]
SLC26A9, Slc26a9	Mouse airway surface liquid	Induction of acidification	[117]
	CFBE41o	Increase in CFTR current	[118]
	Human Asthmatic airway	Overexpressed in cells	[119,120]
	Mouse kidney medullary collecting duct	Increase in arterial pressure	[121]

Abbreviations: Nhe3, sodium hydrogen exchanger 3; CFTR, cystic fibrosis transmembrane conductance regulator; FRTL-5, rat thyroid follicular cell line; CFBE41o, human cystic fibrosis bronchial epithelial cell line.

## Data Availability

The data presented in this study are available on request from the corresponding author. The data are not publicly available.
